# Dual Process for Intentional and Reactive Decisions

**DOI:** 10.1371/journal.pcbi.1003013

**Published:** 2013-04-04

**Authors:** Marie Devaine, Florian Waszak, Pascal Mamassian

**Affiliations:** 1 Université Paris Descartes, Sorbonne Paris Cité, Paris, France; 2 Laboratoire Psychologie de la Perception, CNRS, UMR 8158, Paris, France; University College London, United Kingdom

## Abstract

Efficient cognitive decisions should be adjustable to incoming novel information. However, most current models of decision making have so far neglected any potential interaction between intentional and stimulus-driven decisions. We report here behavioral results and a new model on the interaction between a perceptual decision and non-predictable novel information. We asked participants to anticipate their response to an external stimulus and presented this stimulus with variable delay. Participants were clearly able to adjust their initial decision to the new stimulus if this latter appeared sufficiently early. To account for these results, we present a two-stage model in which two systems, an intentional and a stimulus-driven, interact only in the second stage. In the first stage of the model, the intentional and stimulus-driven processes race independently to reach a transition threshold between the two stages. The model can also account for results of a second experiment where a response bias is introduced. Our model is consistent with some physiological results that indicate that both parallel and interactive processing take place between intentional and stimulus-driven information. It emphasizes that in natural conditions, both types of processing are important and it helps pinpoint the transition between parallel and interactive processing.

## Author Summary

The topic of our study is the interaction between intentional and externally-driven actions. The contemporary literature on motor control in primates clearly distinguishes two neural mechanisms for these two types of actions. We believe that this distinction is artefactual and comes in part from the fact that intentional and externally-driven actions have been studied by different groups of researchers, using different methodologies. In real life however, voluntary planned actions such as making a cup of tea are often interrupted by other actions in response to the outside world. In the present study, we specifically investigate the interaction between intentional and externally-driven actions. We asked participants to anticipate their response to the delayed appearance of a target. We find strong interactions between the prepared action and the target-triggered response: perceptual decisions are quicker when the two actions are congruent. To account for our behavioral results, we propose a computational model that is based on two stages, the first in which external and internal evidence accumulate separately, and the second in which internal processes are modulated by externally-driven ones. This model allows us to establish that intentional and externally-driven actions really interact only in half of the decision making process.

## Introduction

Research on human action control typically distinguishes between two types of action: re-actions issued in response to some external stimulus event and voluntary actions based on an internal decision to act [1], [2]. There has been a long-standing debate on whether these two types of action are controlled by two different brain systems. Some studies using neurophysiological, behavioural or neuropsychological methods suggest that this is indeed the case [3]–[6]; [ for review], [ see Refs. 2,7]. Patients suffering from “utilization behaviour” (UB), for example, show a strong tendency to use objects they spot in the environment without any clear need or purpose [8]. This behaviour has been explained in terms of a lack of inhibition and modulation of the external action system due to damage in the voluntary system [8]. Other studies, however, point to the existence of common control mechanisms. In a recent study [9], for example, participants were asked to prepare and execute left- or right-hand voluntary actions. Occasionally, the voluntary action preparation was interrupted by a stimulus requiring either a left- or right-hand response. The results showed that increased voluntary motor preparation, as assessed by the readiness potential, produced faster stimulus-driven responses on congruent trials (i.e., when participants voluntarily prepared the same hand that was also used in response to the target stimulus) than on incongruent trials. This suggests that voluntary and stimulus-driven actions share some central preparatory mechanisms.

It is evident that voluntary and stimulus-driven action control are at least to some extent based on separate mechanisms, for the simple reason that stimulus-driven but not voluntary action control needs to be linked to the perceptual system. Conversely, it is also obvious that voluntary and stimulus-driven action control are to some extent based on common mechanisms, as it seems undisputed that the most final steps of action execution use the same cerebral structures. Therefore, we propose that an appropriate model to account for voluntary and stimulus-driven actions should be composed of two stages, a first stage where the two types of actions are dissociated and a second stage where they are combined. Thus, the issue is not whether there are one or two systems but rather where the transition between the two stages is. As a consequence, to better understand the interaction between voluntary and stimulus-driven action control, we need to develop tools that embrace the notion of the existence of both differences and commonalities between voluntary and stimulus-driven action and that are capable of pinpointing them.

The goal of the current study is to provide evidence for a two-stage model of action control. Our study is very tightly rooted in a new methodological approach developed by Stanford and colleagues [10]–[12]. Participants are required to initiate a choice response to a left or right target stimulus. Importantly, the target is presented with a variable delay, called *gap*, after participants began to prepare their action. For very short gaps the action is truly stimulus-driven, whereas for long gaps, it is truly intentional, as participants no longer have the time to take the stimulus into account. For intermediate gap durations, the target is possibly able to influence the ongoing voluntary action preparation. This paradigm mimics situations in which we have to anticipate and start preparing an action even before the stimulus the action is supposed to respond to is available, for instance, when a goal keeper has to anticipate where the opponent will shoot the ball and prepare his action even before having any visual cue. The paradigm enables us to investigate whether, and if so, how and from when on voluntary and stimulus-driven action preparation interact.

In one of the studies the present expriment is based on investigated monkeys were required to perform internally/externally chosen left/right saccades [10]. The authors proposed a race-to-threshold model where motor preparation for left/right responses accumulates over time to account for the data [13]–[15]. Models where perceptual evidence accumulates over time have indeed been supported by both behavioral and neurophysiological studies [13]–[17]. However, this particular type of task [10] is not purely perceptual in nature. The participant first starts to prepare an action without the perceptual information being available. It is clear that the evidence accumulated during this stage is not “perceptual”, but rather internal. To this extent, the paradigm is close in spirit to another one used in action control [18].

To capture this aspect of the task, the current research focuses on how, precisely, internal (voluntary) and external (stimulus-driven) accumulation of evidence interact. To model our results we conceptualize internal and external accumulation of evidence as a two-system process having both a separate and a common stage. This hybrid model includes a *transition threshold* below which the signals accumulate separately, and above which they interact (resulting in facilitation in case of congruent and interference in case of incongruent actions). Our experiments also allows us to quantify the relative importance of each stage, thus demonstrating that they are both necessary.

## Results

### Experiment 1: Behavioral results

**Figure 1:**
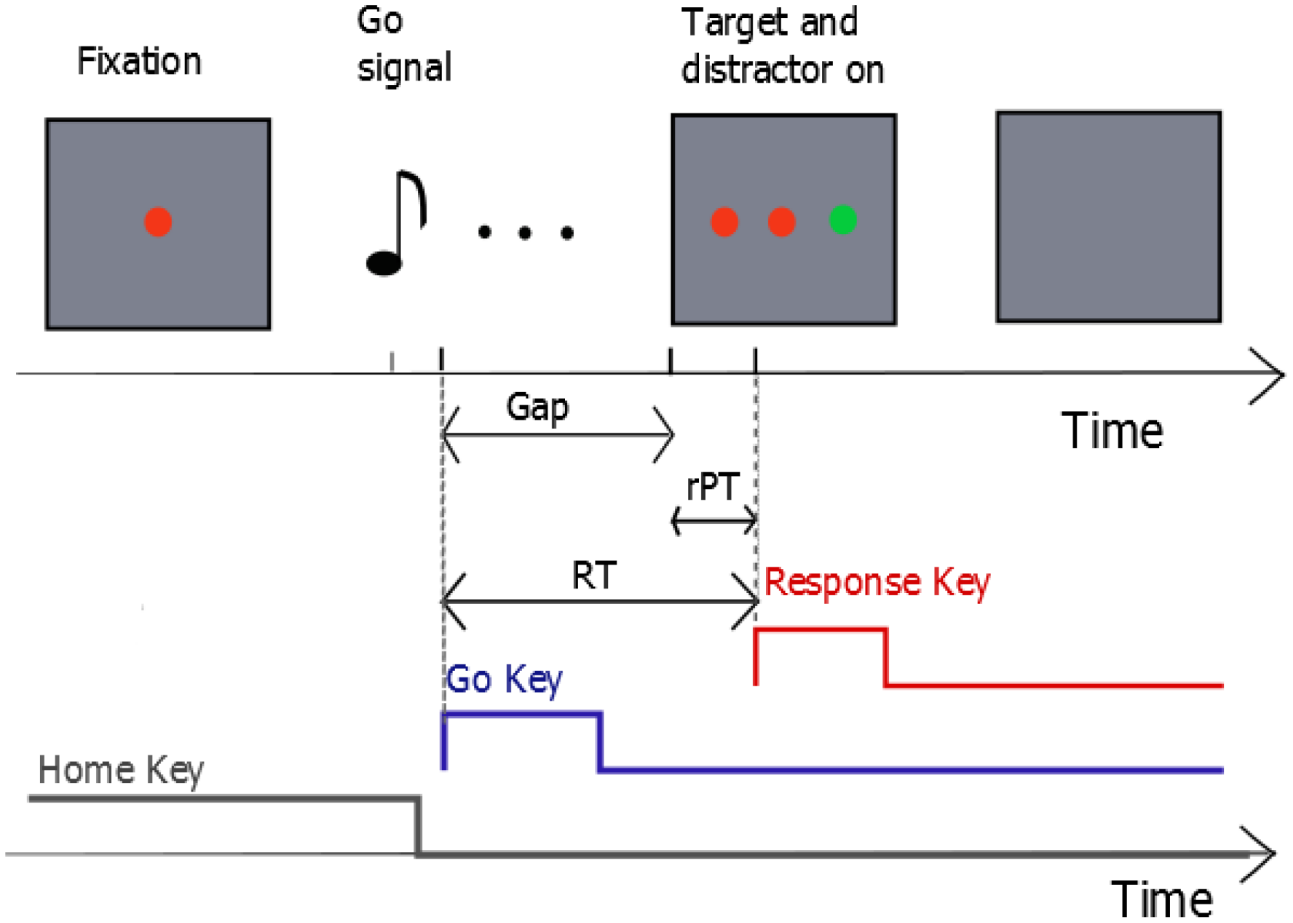
Sequence of trial events. The fixation dot indicates the target color. As soon as the participant hears a sound, the Go Signal, she is required to press one of two Go Keys (‘2’ or ‘8’ on a numeric keypad). Which Go Key to press is indicated by the pitch of the Go signal. After a variable time Gap (0–330ms) that starts when the participant presses the Go Key, a target and a distracter dots appear on either side of the fixation dot. The participant has to initiate her motor response (pressing the key on the left or right of the Go Key to indicate her choice of target location) immediately after pressing the Go Key, even though the actual location of the target is available only after the Gap.

In our task [10], participants were presented with a *Go Signal* followed by the appearance of a *Target Signal* to the left or the right of fixation and a distractor object on the other side (see Fig. 1). The target was defined by its color (red or green) chosen randomly for each trial and indicated to the participant as the color of the fixation point. Participants were instructed to press a key (*Go Key*) in response to the Go Signal and immediately afterwards to initiate a choice response to the side of the target (left or right). The Target Signal was presented after the Go Key with a variable delay called *gap*. Therefore, in trials where the gap is too long, participants had to initiate their choice response even before appearance of the Target Signal. In order to prevent participants from waiting for the presentation of the target and from answering systematically too quickly without taking into account the target, a feedback procedure was introduced that encompassed both speed and accuracy performance (see Methods for details). All participants had an accuracy above chance (mean = 71.5% correct, SD = 5.8%).

**Figure 2:**
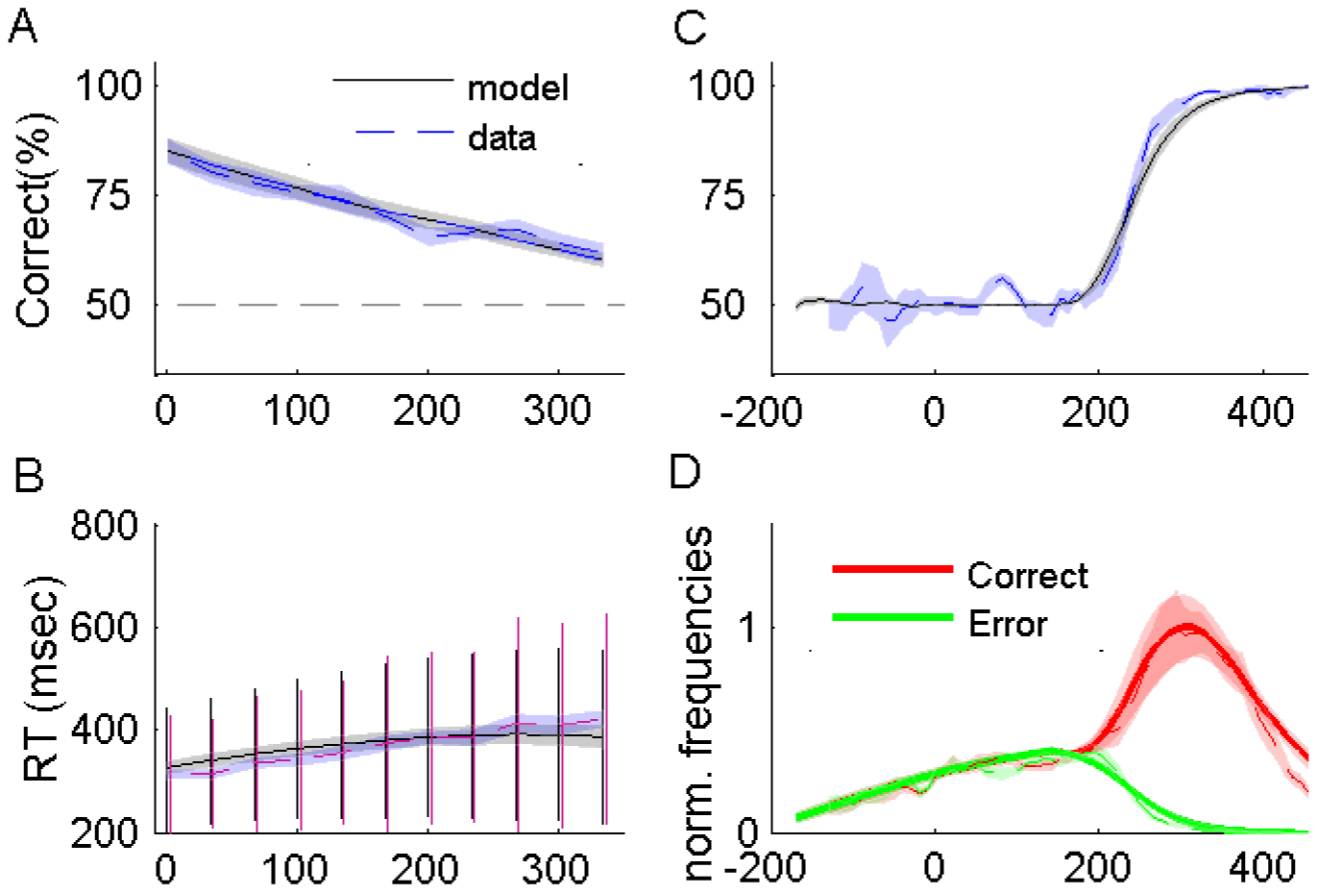
Behavioral results and model performance in Experiment 1. In A, B, and C behavioral data for N = 10 subjects are represented in dashed red lines with the red area corresponding to ±1 s.e. of inter-subjects variability. Results labeled “model” are the averaged fit of the ten subjects using parameters of Table 1. (A) Percentage of correct responses as a function of time gap. (B) Mean reaction time (RT) with standard deviation for the whole data set as a function of time gap. (C) Percentage of correct responses as a function of raw Processing Time (rPT). (D) Normalized frequencies of rPT for correct (red area) and incorrect trials (green area). In C and D bin width is 35 ms. See Fig. S1 in Text S1 for the fit of a typical subject.

We present data pooled across all ten participants. As anticipated, accuracy significantly decreased as the gap increased (F(10,99) = 9.4, p<0.0001), simply because participants did not have any opportunity to revise their initial decision if the gap was too long. In addition, Response Times (RT), that is, the time between the Go Key press and the choice response, increased gradually with increasing gap (F(10,99) = 6.6, p<0.0001). However, the slope of RT increase with gap was less than one for all participants (mean estimated slope = 0.34, maximum of the upper bound of the 95% individual confidence interval = 0.71) indicating that participants possibly waited for the target to appear on some trials but not all (Fig. 2 A–B).

A key variable of the analysis is the *raw Processing Time* (rPT) introduced by Stanford and colleagues (10). The rPT is the time during which the target information was available before the choice response was carried out. More precisely, the raw processing time is defined as: rPT = 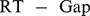. Thus, positive values correspond to a choice response after target onset, and negative values correspond to a response before target onset. The percentage of correct choice responses increased sharply with rPTs (Fig. 2C): under a critical value of rPT (202 ms±.4, s.e. computed with a Bootstrap procedure) responses were given at random whereas above this value accuracy reached quickly 100%. Fig. 2D shows the normalized distribution of the rPTs separately for correct and error trials. The distribution of correct choice responses looks like the superposition of two component distributions, one identical to the rPT distribution in the case of erroneous responses, and one specific to correct responses. This component reflects actions carried out without perceptual information being taken into account (because the rPT was too short). The second component of the correct distribution, corresponding to longer rPTs, reflects actions in response to the target.

### Modeling

**Figure 3:**
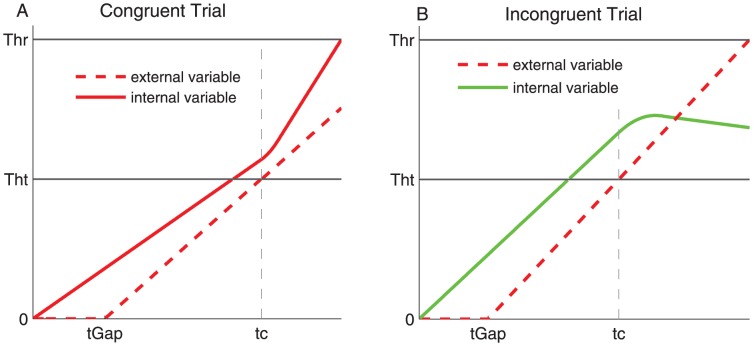
Illustration of congruent and incongruent trials in the Hybrid model. The internal variable starts accumulating as soon as the Go Key is pressed whereas the external variable starts accumulating after 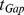 which varies across trials. During the first stage, the two variables accumulate independently. As soon as both variables have entered the common part (at 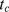), the external variable starts influencing the internal one: for congruent trials (A) the internal accumulation rate increases in a smooth way to reach the sum of the initial internal rate and the external rate. (B) In incongruent trials the internal variable is inhibited and its rate decreases to reach the difference between the initial internal rate and the external rate. Note that congruent trials are always correct, whereas incongruent trials can be incorrect.

We propose a hybrid model in which the decision of a particular choice response is the result of a two-stage race between an internal variable that codes randomly for one or the other response and an external variable that codes for the target side. During the first stage of the race, the two variables accumulate independently, each at a constant rate drawn from a lognormal distribution of a certain mean and variance. The internal variable starts accumulating as soon as the Go key is pressed while the external variable starts accumulating only at the appearance of the target (see Fig. 3). Indeed, until target presentation the participant has no sensory information to rely on.

It is only after both variables crossed a first threshold, the *Transition Threshold*, that the common stage starts where the external variable influences the internal one. We distinguish congruent from incongruent trials depending on whether internal and external variables code for the same or different responses. In congruent trials, the external facilitates the internal variable. In contrast, in incongruent trials, the external inhibits the internal variable. The first variable to cross the second threshold, the *Response Threshold*, triggers execution of the action it codes. In our task the action is most often triggered by the internal variable because the internal variable always starts accumulating first. However, it can happen (especially for short gaps) that the external crosses the second threshold before the internal crosses the first threshold. In that case that resembles a traditional reaction time experiment, the action is purely triggered by the external stimulus.

Please notice that the RT distributions are necessarily a mixture of congruent and incongruent trials (where internal and external variables coded for the same or different responses). However, the congruency of the two variables is in principle unknown to the experimenter, as the internal decision of the participant is not known. To deal with this problem, we present a method to estimate the distributions for the two types of trials (congruent and incongruent) in the second Experiment.

The model includes seven parameters: the means 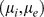 and standard deviations 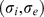 of the lognormal distribution of the internal and external accumulation rates, the Transition Threshold 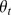 (the Response Threshold is set arbitrarily to 1000, all other parameters being scaled relative to this value),the acceleration factor *A* that represents the time needed for the external variable to fully influence the internal one, and an execution delay 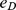.

Table 1 presents the values of the parameters corresponding to the best fit for each participant obtained by maximizing the likelihood of the rPT distribution (the averaged model fit is represented as continuous lines in Fig. 2).

**Table 1:** Model parameter values in Experiment 1.

Participant	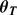	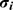	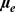	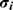	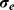	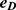 *(ms)*	*A*
1	552	1.4	1.4	1.2	0.3	125	21
2	493	1.6	1.0	1.7	0.4	129	11
3	404	1.4	1.1	0.8	0.5	120	76
4	428	1.1	0.9	1.3	0.4	111	81
5	528	2.1	1.4	1.9	0.4	130	10
6	559	2.3	1.3	1.8	0.4	127	21
7	502	1.5	1.5	0.7	0.4	141	73
8	491	1.0	1.7	0.8	0.4	138	8
9	498	1.0	1.4	0.6	0.3	121	106
10	427	1.4	1.2	1.2	0.5	173	12

Because our task is similar to that used by Stanford et al. [10], we can compare the performance of our hybrid model with their model, which is a single race-to-threshold between two decision variables, representing right and left choices. The two variables start accumulating with randomly drawn rates, then, after the gap, color discrimination affects the decision process by accelerating the variable representing the target side and decelerating the variable of the distractor side. Without affecting the spirit of their model, we used a slightly simplified version that contained only eight parameters rather than eleven (see Text S1). We also compared our model to a simpler version of it that was used in another task [19], a version of the drift diffusion model [13] adapted to our task and an Ornstein-Uhlenbeck process [20]. In this model there is only an independent race stage without then transition threshold. We compared the models at the group level [21] using BIC (Bayesian Information Criterion) and AIC (Akaike Information Criterion) for each model and each subject as a measure of evidence. The performance of our model was superior to the other models: the exceedance probability [21], i.e. the probability that our model was the more frequent in our population of subject, was greater than .95 both for the AIC criteria and the BIC criteria (see Table S1, S2 in Text S1 for the individual AIC and BIC values).

### Experiment 2: Introducing a bias to estimate the congruent/incongruent distributions

Our model is based in part on the accumulation of evidence of an internal variable that is aimed arbitrarily to one side or the other. When this internal variable crosses the transition threshold, it starts to be affected by the stimulus-driven process. At that point, the stimulus-driven process can either facilitate or inhibit the intentional process, depending on whether the initial decision is congruent or incongruent with the stimulus. Unfortunately, in our first experiment, there is no way to analyze separately the congruent and incongruent conditions, simply because we do not know what the initial decision was. Rather than asking explicitly to the participant what his initial decision was at the end of each trial, we slightly modified the design of our first experiment. In order to estimate the rPT distributions separately for congruent and incongruent trials, we introduced a frequency bias in the second experiment. Unbeknown to the participants, the probability of the target being on one or the other side depended on the pitch of the Go signal. We refer to the side that had the higher probability (65%) to be the target side as the “*more frequent side*”.

Eleven out of fourteen participants expressed the expected response bias for the more frequent side. The bias ranged from 55% to 75% (mean = 65.3%, SD = 6%). The data of these eleven participants were analyzed together. The remaining three subjects were excluded from the analysis either because the bias was too extreme or absent. The basic features of the results of Experiment 2 are identical to Experiment 1 (see Fig. S2 in Text S1).

We adapted our hybrid model to this second experiment. In the model described earlier, both sides were chosen with the same *a priori* probability. The response bias in the current experiment is modeled by introducing a bias α on the choice of the internal variable for the more frequent side. If participants did adapt perfectly to our experimental setup, the value of α should be 0.65. The new hybrid model including this additional bias parameter was adjusted to the individual data; the average of the best fits for each subject is superimposed on the curves in Fig. S2 in Text S1 and their parameters are shown in Table 2.

**Table 2:** Model parameter values in Experiment 2.

Participant	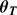	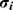	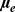	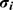	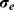	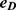 *(ms)*	*A*	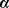
**1**	427	1.0	1.3	0.4	0.4	102	5	0.66
**2**	584	1.6	1.0	1.2	0.3	130	56	0.62
**3**	830	1.3	1.1	0.3	0.3	109	81	0.74
**4**	341	0.6	1.7	0.8	0.8	103	62	0.71
**5**	623	1.2	0.6	0.8	0.3	109	31	0.58
**6**	464	1.1	1.5	1.2	0.6	102	28	0.73
**7**	300	0.5	1.2	0.6	0.3	102	12	0.62
**8**	338	0.9	2.1	0.6	0.5	102	8	0.60
**9**	525	1.0	1.0	1.3	0.4	92	103	0.46
**10**	438	1.1	1.9	2.1	0.2	102	93	0.56
**11**	382	1.6	1.3	0.6	0.4	140	81	0.53

**Figure 4:**
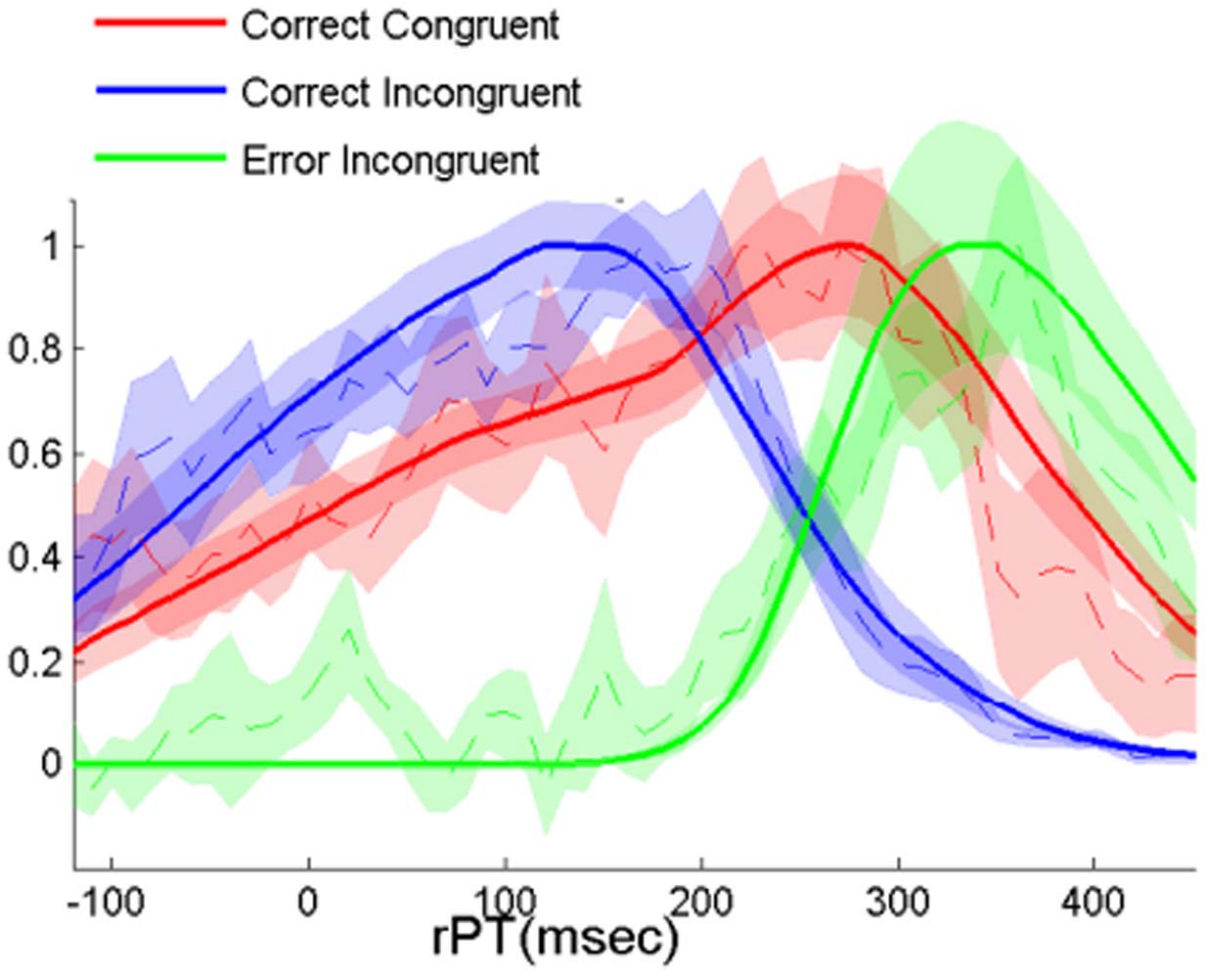
Congruent and incongruent distributions. Model and behavioral rPT distributions for congruent (in blue) and incongruent trials (green for correct and red for error trials), for N = 11 participants. Data curves (dashed lines) are obtained from the distributions for more frequent size and less frequent size via a linear transformation. The colored area around the dashed lines represent +/− s.e. of inter-subject variability. Model curves are the averaged individual fits of the eleven subjects using the parameters given in Table 2. Bin width is 35 ms. (See Fig. S2 in Text S1 for the rest of model and behavioral performance).

In the model, the external and the internal variables either code for the same side or they code for different sides, thereby defining congruent and incongruent trials. As a consequence, rPT distributions can be seen as the mixtures of two distributions corresponding to congruent and incongruent trials. Because of the bias in the model, the proportion of correct trials on the more frequent side is α for congruent trials and (1- α) for incongruent trials. On the less frequent side, the proportion of correct trials is (1- α) for congruent trials and α for incongruent trials. Using a linear transformation accounting for the bias we can thus estimate the rPT distributions for correct congruent and incongruent trials (Fig. 4). The figure reveals that target information needs more time to influence incongruent trials than congruent ones, notably the incongruent model distribution peaks 48 ms later than the congruent one. Keeping in mind that the external variable always codes for the correct side, error trials are thus necessarily incongruent trials. Therefore, the rPT distribution of error incongruent trials can be computed by taking all the error trials together.

## Discussion

The aim of the present study was to assess and model the interaction of stimulus-driven and voluntary action control. Participants were presented with the stimulus of a speeded perceptual discrimination task during the preparation of a voluntary action. Depending on the time gap between the beginning of the trial and the presentation of the target stimulus, voluntary action preparation was differently advanced when the target appeared, thus, enabling us to trace the effect of voluntary action preparation on stimulus-driven behavior.

In the first experiment, a good predictor of accuracy was the raw processing time rPT, which is the time during which the target information was available before the stimulus-driven choice response was executed. For short rPTs, response accuracy was at chance. However, as soon as rPTs exceeded a critical value, it rapidly increased to reach ceiling performance. In other words, for short rPTs participants performed in an internal mode, choosing the response at random. It is only for longer rPTs that they take target information into account. The transition between purely internal and based-on-evidence modes was very fast although we analyzed the pooled data set. This shows that both the critical rPT value and the fast transition are quite robust among participants, since inter-subject variability would smooth the curve. Overall, our behavioural results are thus very much in line with the two monkeys' behaviour in in Stanford et al.'s study [10]. A critical difference in the behavioural pattern concerns the RT that are longer in our study, which is consistent with the fact that saccades initiations (as in the monkey study) are much faster than manual response [22].

To account for the shape of the distribution of rPTs, we suggested a physiologically founded model, the hybrid model, which is in essence a two-stage race model between a variable of the external and a variable of the internal action system. Our model is “hybrid” in two ways. First because it incorporates two systems, external and internal, secondly because the race has two stages: a stage at which evidence in the two systems accumulates independently and a later stage at which evidence accumulation in the two systems interacts. The distinction between internal and external systems follows the literature of stimulus-driven and voluntary action [5], [6], [8], [18], [23]. A recent study [24] that combines functional magnetic resonance imaging (fMRI) and pattern recognition shows that this distinction between two systems is also relevant in the framework of perceptual decision making. Indeed, this study shows that decisions regarding highly visible stimuli are predicted by visual brain areas, whereas it is not the case for low visibility when participant decisions are at chance level. In the latter case, the precuneus, which has been shown to encode “free” decisions [25], is a good predictor of the final choice. However, our hybrid model does not reduce to a simple “switch” between a guessing system and a perceptual decision system since it has two stages. This fits with the growing body of evidence that information processing is not purely sequential [26]. The distinction of these two stages is based on the fact that the external and the internal action routes are necessarily separated at least up to some point, since the external system needs to make contact to visual processing, and probably merged from that point on. As outlined above, the hybrid model fits our data very well, better than models that only incorporate a single system or a single stage [10], [13], [19], [20].

Since, in the hybrid model, accumulation of evidence occurs in two systems, an external and an internal system, a particular trial can be congruent or incongruent, external and internal variables either coding for the same response or for different responses, respectively. The congruency of a trial determines how the two variables interact in the second stage of processing. In congruent trials the internal variable is facilitated, whereas in incongruent trials, it is inhibited. As a consequence rPTs of the based-on-evidence component should be longer in incongruent than in congruent trials. To test our model it was therefore essential to have rPT distributions separately for congruent and incongruent trials. However, note that it is difficult to know whether a particular trial is congruent or incongruent, since this information requires access to the variable coding for the internally chosen action. One solution to this problem is to ask participants at the end of the trial which action they initially prepared and, using some brain imaging technique (e.g., EEG), to cross-check the introspection of the subject (e.g., by means of ERPs; see Ref. [9]). Here we chose a different approach: instead of relying on participants' introspection, we decided to deduce the congruent/incongruent distributions analytically in the second experiment. To do so, we made the probability of the target being presented on one or the other side depends on the pitch of the Go signal. Due to the induced response bias α, the rPT distributions of correct trials result from a proportion of α congruent and (1-α) incongruent trials for the more frequent response and from a proportion of (1-α) congruent and α incongruent trials for the less frequent response. Thus, in Experiment 2, we were able to estimate the rPT distributions for correct congruent and incongruent trials by means of a linear transformation; this was not possible in Experiment 1, where α = 1- α, making a linear transformation impossible. The results show that “based-on-evidence” incongruent trials correspond to longer rPTs than congruent trials, suggesting interaction between the internal and the external system. This is in agreement with the results reported in a recent study [9] in which the authors found reaction times to be longer for incongruent than for congruent trials.

The hybrid model also allows us to estimate the relative amount of processing required in the two stages (independent and common), as the relative importance of the two stages is captured by the value of the transition threshold. Experiments 1 and 2 revealed that a considerable amount of processing is done in both stages so that both stages are necessary. In both experiments, the mean threshold was approximately around 50% of the overall evidence accumulation, showing that about half of processing is done separately and the other half in a combined way. Moreover, the individual participant variability is well capture by our model.

In most previous modeling attempts, only one stage is present: either the two variables accumulate independently of each other or they interact during the whole race. For instance, a recent study [27] introduced several race-to-threshold units to model a Go-No Go task. If the variable in the Stop unit reaches its threshold before the variable in the Go unit, it cancels the race of the later (see also Ref. [28]). It is important to keep in mind that even though they are simultaneous, the races in the different units stay independent of each other: the rate of accumulation of one variable is not influenced by any other. In other models, like the leaky accumulator [15], variables inhibit each other during the whole process. For instance, on the late distractor effect in saccadic inhibition [29] has been successfully modeled by a dual input combined with mutual.

To our knowledge, only one recent study has also proposed a two-stage diffusion model. In an effort to account for behaviors that resemble a change of mind in the course of a manual action, Resulaj and colleagues [30] introduced a change of mind bound and a change of mind deadline to the original diffusion process. This model can be seen as the concatenation of two diffusion processes where a second diffusion can be initiated after a first decision. In contrast, our model clearly distinguishes a first stage where two variables are processed independently from a second stage where these variables interact. We believe that models based on two stages of processing will be inspiring for future attempts of modeling race-like phenomena.

## Methods

### Ethics statement

Participants were voluntary and gave their informed consent. Research was approved by the Ethics committee for biomedical research (CERB) Ile de France II.

### Participants

Ten healthy participants with normal or corrected-to-normal vision participated in the first experiment (6 males, 4 females; mean age: 23 years and 3 months, SD: 1 year and 3 months). Fourteen healthy participants with normal or corrected-to-normal vision participated in the second experiment (8 male, 6 female; mean age: 23 years and 5 months, SD: 2 years and 1 month). All subjects were naive with respect to the goal of the experiment.

### Material

Visual stimuli were presented on a computer screen LIYAMA HM 903 DTA (19 in). The experiment was controlled using Matlab and the Psychtoolbox [31], [32]. Visual stimuli were two colored dots (target and distractor), one red and one green presented each on one side of a third dot. The color of the central dot varied randomly from trial to trial. It was either red or green. Each dot had a radius of 44 pixels and the distance between the central dot and the peripheral dots was of 134 pixels, viewing distance was 50 cm. An auditory signal of 50 ms duration indicated the beginning of the task (Go signal). Its pitch varied randomly on each trial. It was either high (1500 Hz) or low (600 Hz).

### Task details

#### Experiment 1

From the beginning of the trial and until the Go signal, the participant was required to keep the “Home Key” pressed (Key 5 of the numeric keys of a standard keyboard). Before the (Go signal) was presented, a dot appeared in the center of the screen; its color was either red or green: this indicated the color of the future target. After a delay randomly drawn from a normal distribution of mean 1.6s and standard deviation 0.1s, an auditory stimulus, the Go signal, was presented. Its pitch was either high or low, thereby indicating the part of the numeric keypad the participant have to use in the current trial. Just after the sound had been presented, the participant was required to press one of the two Go Keys: if the pitch was high, the participant had to press Key 8 (Go Key 1), if it was low s/he had to press Key 2 (Go Key 2). Immediately after having pressed the Go key, the participant had to initiate a motor action either to press the Key on the right (9 and 3 for Go Key 1 and 2 respectively) or on the left of the Go Key (7 and 1 for Go Key 1 and 2 respectively), although at this point the identities of the target and distracter are still unknown. These are revealed later after a time gap that was drawn from a uniform distribution and varied between 0 and 330 ms (measured from the moment the Go Key was pressed on) when a red and a green dot appeared on either side of the central dot. The participant was required to press the key on the side of the target.

The participant received feedback every 20 trials in the form of 2 scores: speed points corresponding to the number of trials over the 20 last trials for which the responses was quick enough (the delay between the auditory signal and the response had to be less than 850 ms) and accuracy points corresponding to the number of correct responses over the last 20 trials.

The experiment was divided into two sessions of about 45 min each. The first session consisted in a training phase and the first part of the test phase. The second session consisted in the second part of the test phase. During the first part of the training phase, only the speed score was given as a feedback, whereas during the second part of the training phase both scores were provided.

#### Experiment 2

Experiment 2 was identical to Experiment 1, except that a bias was introduced. Unbeknown to the participant, the probability of the target being left or right of the central dot depended on the pitch of the Go signal. For half of the participants, after a high pitch, the target color appeared more often on the right side (in 65% of cases); after a low pitch, the target color appeared more often on the left side (65%). For the other half it was the other way round. In the analysis we refer to “more frequent” side and “less frequent” side collapsing data across the sounds and the two groups of participants. For instance, in the first group of participants, the “more frequent side” side refers to right if the sound was high pitched and left if the sound was low pitched.

### Data analysis

In the two experiments we analyzed the pooled data of all subjects excluding trials in which reaction time (RT) deviated more than 4 standard deviations from the mean. We fitted the Hybrid model, the single independent stage model [19], the diffusion model [13], the Ornstein-Uhlenbeck process [20] and Stanford et al.'s model [10] by maximizing the likelihood of the rPT distribution for each subject independently. We used the same procedure in the second experiment to fit the Hybrid model with the internal bias as an additional parameter. In the second experiment, we inverted the matrix 
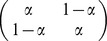
 to find the weights of the renormalized distribution of rPT for congruent and incongruent trials as a mixture of the renormalized distribution of rPT for “more frequent” and “less frequent” trials. We set α = .65, the true bias.

### Model description

In the Hybrid model, the decision process is split into two stages. During the first stage, internal and external variables accumulate without interacting until they reach their transition threshold and enter the common part. In the common part, internal and external variables interact in a way that depends on the congruency of internal and external variables. The label (i.e., left or right) of the internal variable is drawn randomly from a Rademacher distribution. The initial accumulation rate 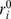 of the internal variable during the first stage is drawn from a lognormal distribution of mean 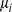 and standard deviation 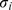, i.e.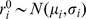. Similarly, the accumulation rate 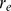 of the external variable during the first stage is drawn from a lognormal distribution of mean 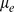 and standard deviation 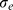, i.e. 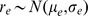. The internal variable starts accumulating as soon as the go Key has been pressed at time 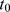 whereas the external variable starts accumulating as soon as the cues have appeared on the screen, i.e. at time 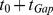 (we consider no afferent delay for sake of simplicity). We can thus write the value of the variables at each time 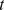 during the first phase: 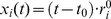 and 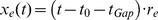 (for 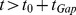).

Once both variables have reached the transition threshold at time 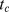, where 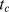 is the first 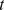 to verify 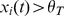 and 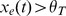, the accumulation rate of the internal variable is influenced by the external variable. More precisely, we have a distinction between congruent and incongruent trials:

- In congruent trials, the final internal rate will be the sum of the internal rate and external rate: 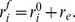

- In incongruent trials, the final internal rate will be the difference of the internal rate and of the external rate: 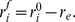

The transition between 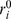 and 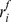 is continuous and follows the differential equation:

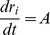
 for congruent trials

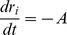
 for incongruent trials

The first variable to reach the common threshold determines the side chosen, and the time at which it crosses the response threshold plus the execution delay (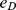) gives the reaction time.

### Models comparison

We compared our Hybrid model with three other models based on the rPT distribution obtained for each participant in the first experiment. We used simulations to compute the likelihood of each participant distribution of rPT under each model and sets of parameters (see Text S1). The best set of parameters was obtained by maximizing the likelihood (*L*) for each model, then we computed the AIC and BIC for each subjects using the following formulae for a model using *p* parameters, and *n* observed rPTs:
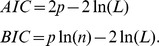



## Supporting Information

Text S1 Text S1 provides details about the model comparison we perform, and displays the corresponding fits of a typical participant.
(DOCX)
