# Supplementary material for: Dual Process for Intentional and Reactive Decisions
Source: PLoS Comput Biol. 2013 Apr 4;9(4):e1003013. doi: 10.1371/journal.pcbi.1003013 (PMC3617197; doi:10.1371/journal.pcbi.1003013)
Supplement: Text S1 — provides details about the model comparison we perform, and displays the corresponding fits of a typical participant. (DOCX) [file pcbi.1003013.s001.docx]

**Text S1**

1. **Model Comparison**
   1. Competing models

We compared the performance of our model to four other models: a diffusion model (1), an Ornstein-Uhlenbeck process (2), an independent race model (3) and Stanford et al.’s model (4).

The first model we include in our model comparison is a simple model in line with modeling perceptual decision making with diffusion processes. In this diffusion model, a single decision variable measures the accumulated evidence in favor of “right”. The process starts at zero between the threshold for choosing “right” (T) and for choosing “left” (-T). During the gap, this process follows a random walk with a drift rate equals to zero and with variance $\sigma$. As soon as the target is presented, the mean of this process is set to $\mu$. As soon as one of the thresholds is crossed, the response is triggered and executed with some delay $e_{D}$. This model is thus the simplest with only three parameters: $\mu$, $\sigma$, and $e_{D}$.

We also considered a more sophisticated model, using the Ornstein- Uhlenbeck (OU) process that is an extension of the diffusion process but with a decay parameter $\lambda$ on top of the other parameters $\mu$, $\sigma$, and $e_{D}$. The addition of this free parameter did not improve much the fit.

The second model to which we compared our model has been proposed by Adam et al. (1) and constitutes a simpler version of our Hybrid model with only the independent stage. In this independent race model, two decision variables, an internal one and an external one rise to a threshold at a constant rate drawn from a log-normal distribution. The parameters are thus the same than in our Hybrid model except for the transition threshold and the acceleration factor that are not present in this simpler version. This model includes five parameters: the mean $\left( \mu_{i},\mu_{e} \right)$ and standard deviations $\left( \sigma_{i},\sigma_{e} \right)$ of the lognormal distribution of the internal and external accumulation rates and the execution delay $e_{D}$.

The third one is the model proposed by Stanford et al. (Ref. 3). In the form given by the authors, the total number of parameters of their model was 11:

1.$r_{G}$ the mean of first stage accumulation rate

2. $\sigma_{G}$the standard deviation of first stage accumulation rate

3. $\rho_{G}$ the correlation between target and distracter accumulation rate (in the second

stage)

4. $r_{T}$ the target asymptotic accumulation rate

5. $r_{D}$ the distracter asymptotic accumulation rate

6. $T_{s}$ the sensory discrimination time (ms), i.e. the duration of the acceleration stage

7. $T_{nd}$the non-decision time (i.e. total delay) (ms)

8. $\sigma_{Tnd}$ the standard deviation of non-decision time (ms)

9. $T_{i1}$ the start of interruption time relative to cue (ms)

10. $T_{i2}$ the end of interruption time relative to cue (ms)

11. $P_{conf}$the probability of confusion between target and distracter.

Because it seemed to us that the three last parameters had been introduced in the model to account for particular features of the data of one monkey of their study (in particular a dip that we don’t find in our data), we set $T_{i1}$ = $T_{i2}$ =0 and $P_{conf}$=.001 for the sake of simplicity and to limit the number of free parameters.

- 1. Procedure

To compute the likelihood associated with the rPT distribution of a participant under a given model ($m$) and a given set of parameter ($\theta$), we first simulated the distribution of rPT obtained under this particular model ($m,\theta$). For each rPT value $t$ of this participant, we computed the probability (given the model distribution) $p(t|m,\theta)$ to have observed this data point. The total likelihood L was then obtain as the product of the likelihood of each observed rPT, ${L=\Pi}_{t}p(t|m,\theta)$.

This procedure was first applied to a grid of parameter for each model and each subject. Then, we used fminsearch (in matlabWorks) to further optimize the parameters around the best parameters of the grid.

| Participant | Diffusion model | OU process | Independent race model | Stanford et al. model | Hybrid model |
| --- | --- | --- | --- | --- | --- |
| 1 | 1.361E+04 | 1.361E+04 | 1.340E+04 | 1.339E+04 | 1.334E+04 |
| 2 | 1.389E+04 | 1.379E+04 | 1.369E+04 | 1.383E+04 | 1.369E+04 |
| 3 | 1.334E+04 | 1.334E+04 | 1.335E+04 | 1.323E+04 | 1.324E+04 |
| 4 | 1.368E+04 | 1.364E+04 | 1.357E+04 | 1.346E+04 | 1.347E+04 |
| 5 | 1.393E+04 | 1.383E+04 | 1.364E+04 | 1.367E+04 | 1.360E+04 |
| 6 | 1.407E+04 | 1.407E+04 | 1.380E+04 | 1.384E+04 | 1.378E+04 |
| 7 | 1.322E+04 | 1.322E+04 | 1.328E+04 | 1.314E+04 | 1.314E+04 |
| 8 | 1.242E+04 | 1.242E+04 | 1.246E+04 | 1.247E+04 | 1.239E+04 |
| 9 | 1.267E+04 | 1.267E+04 | 1.271E+04 | 1.261E+04 | 1.261E+04 |
| 10 | 1.338E+04 | 1.338E+04 | 1.337E+04 | 1.330E+04 | 1.329E+04 |

**Table S1: AIC (Akaike Information Criterium) values of the four models for each participant in Experiment**

| Participant | Diffusion model | OU process | Independent race model | Stanford et al. model | Hybrid model |
| --- | --- | --- | --- | --- | --- |
| 1 | 1.363E+04 | 1.362E+04 | 1.343E+04 | 1.343E+04 | 1.337E+04 |
| 2 | 1.390E+04 | 1.380E+04 | 1.372E+04 | 1.386E+04 | 1.372E+04 |
| 3 | 1.335E+04 | 1.336E+04 | 1.337E+04 | 1.327E+04 | 1.327E+04 |
| 4 | 1.370E+04 | 1.365E+04 | 1.359E+04 | 1.350E+04 | 1.350E+04 |
| 5 | 1.395E+04 | 1.384E+04 | 1.366E+04 | 1.371E+04 | 1.363E+04 |
| 6 | 1.409E+04 | 1.410E+04 | 1.382E+04 | 1.388E+04 | 1.382E+04 |
| 7 | 1.323E+04 | 1.324E+04 | 1.330E+04 | 1.318E+04 | 1.317E+04 |
| 8 | 1.244E+04 | 1.244E+04 | 1.248E+04 | 1.251E+04 | 1.243E+04 |
| 9 | 1.268E+04 | 1.269E+04 | 1.273E+04 | 1.265E+04 | 1.265E+04 |
| 10 | 1.339E+04 | 1.340E+04 | 1.339E+04 | 1.334E+04 | 1.333E+04 |

**Table S2: BIC (Bayesian Information Criterium) values of the four models for each participant in Experiment 1**

1. **Supplementary Figures**

**
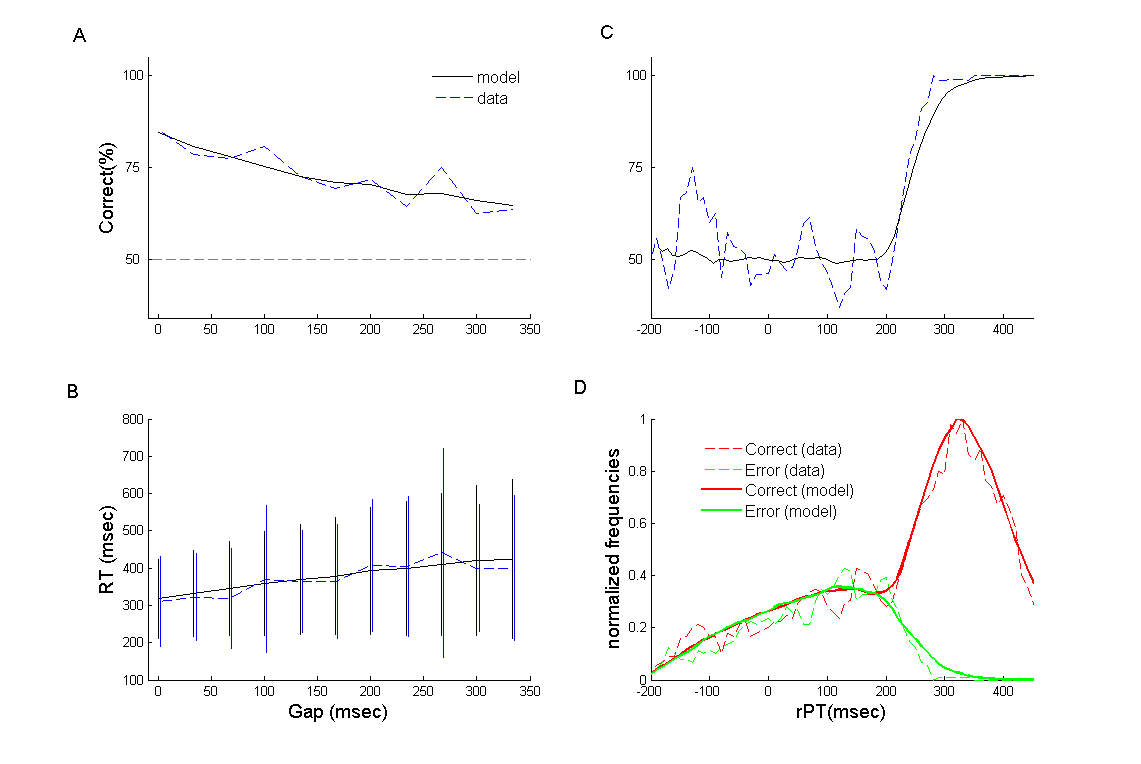
**

Figure S1: Experiment 1, Individual fit of Participant 1 for the Hybrid model. In A, B and C behavioral data are represented in dashed blue lines. Results labeled “model” are from simulated trials generated with parameters for Participant 1 given in Table 1 of the main text. (A) Percentage of correct responses as a function of time gap. (B) Mean reaction time (RT) with standard deviation for the whole data set as a function of time gap. (C) Percentage of correct responses as a function of raw Processing Time (rPT). (D) Normalized frequencies of rPT for correct (red area) and incorrect trials (green area). In C and D bin width is 35 ms.

**
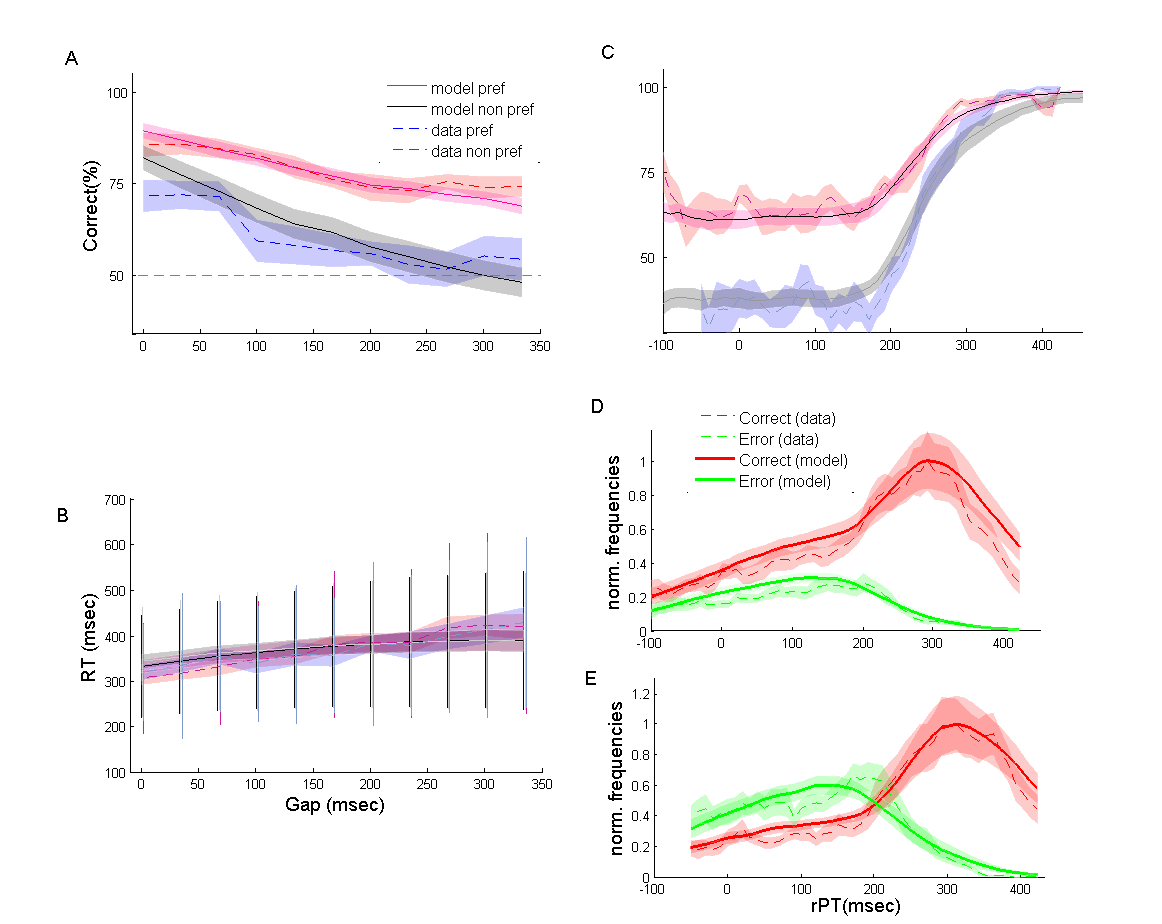
**

Figure S2: Behavioral and Hybrid model performance in Experiment 2. Behavioral data are represented in dashed lines, model fits are in solid lines. In A, B C and D the colored area corresponding to ± 1 s.e. of inter-subjects variability. Results labeled “model” are an average of simulated trials generated with the individual fits (A) Percentage of correct responses as a function of time gap for more and less frequent responses. (B) Mean and standard deviation of reaction time (RT) as a function of time gap for more and less frequent side. (C) Percentage of correct responses as a function of rPT for more and less frequent responses. (D) Normalized frequency of rPT for correct (red lines) and incorrect trials (green lines) for the more frequent side. (E) Normalized frequency of rPT for correct and error trials for the less frequent side. In C, D and E bin width is 35 ms.

**
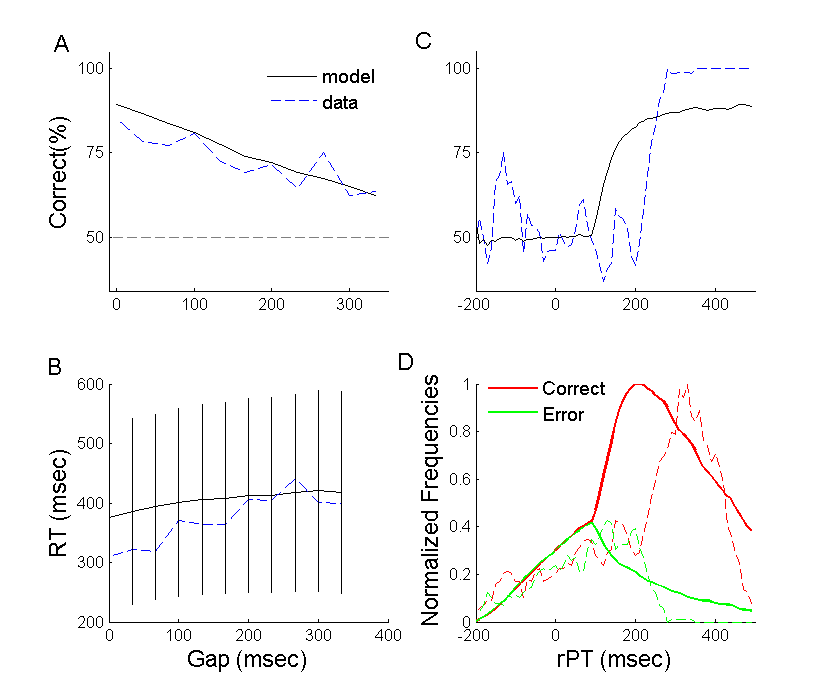
**

**Figure S3: Experiment 1, Individual fit of Participant 1 for the Diffusion model.** In A, B and C behavioral data are represented in dashed blue lines. Results labeled “model” are from simulated trials generated with best fitting parameters for Participant 1. (A) Percentage of correct responses as a function of time gap. (B) Mean reaction time (RT) with standard deviation for the whole data set as a function of time gap. (C) Percentage of correct responses as a function of raw Processing Time (rPT). (D) Normalized frequencies of rPT for correct (red area) and incorrect trials (green area). In C and D bin width is 35 ms.

**
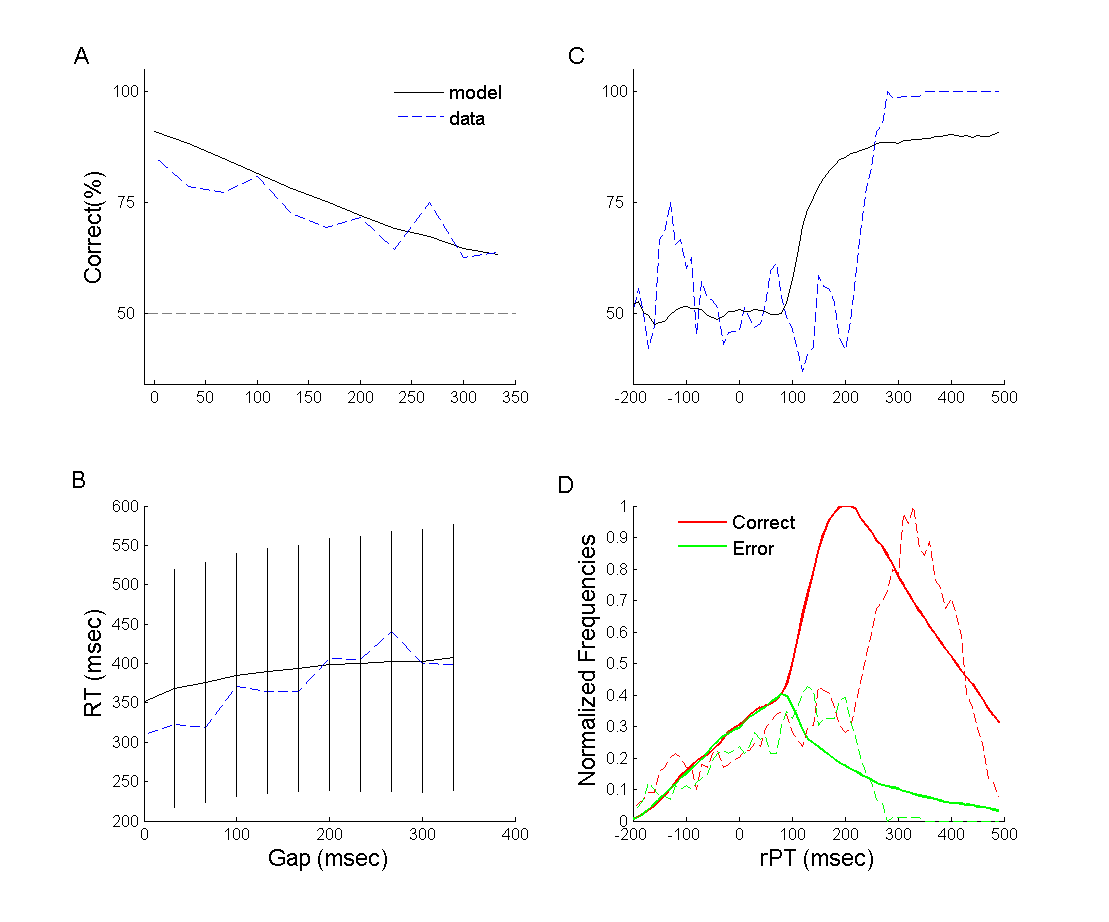
**

**Figure S4: Experiment 1, Individual fit of Participant 1 for the OU model.** In A, B and C behavioral data are represented in dashed blue lines. Results labeled “model” are from simulated trials generated with best fitting parameters for Participant 1. (A) Percentage of correct responses as a function of time gap. (B) Mean reaction time (RT) with standard deviation for the whole data set as a function of time gap. (C) Percentage of correct responses as a function of raw Processing Time (rPT). (D) Normalized frequencies of rPT for correct (red area) and incorrect trials (green area). In C and D bin width is 35 ms.

**
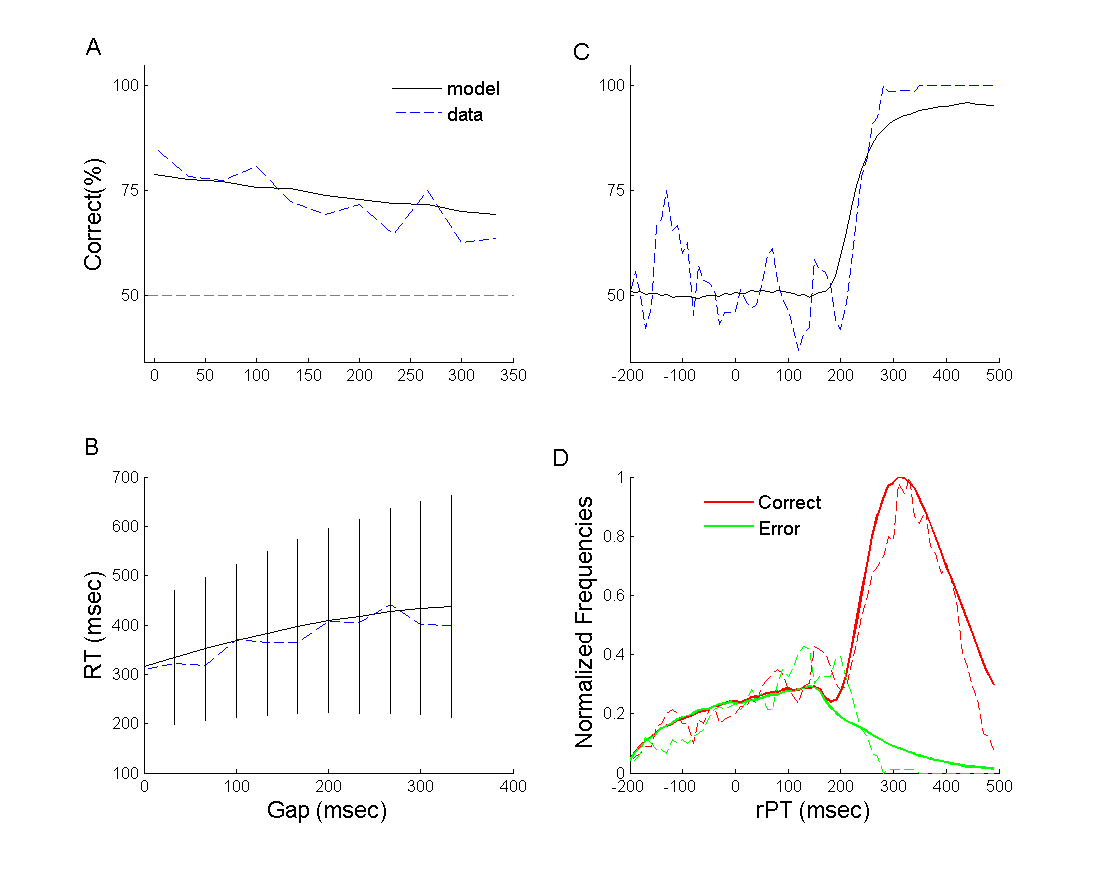
**

**Figure S5: Experiment 1, Individual fit of Participant 1 for the Adam et al model.** In A, B and C behavioral data are represented in dashed blue lines. Results labeled “model” are from simulated trials generated with best fitting parameters for Participant 1. (A) Percentage of correct responses as a function of time gap. (B) Mean reaction time (RT) with standard deviation for the whole data set as a function of time gap. (C) Percentage of correct responses as a function of raw Processing Time (rPT). (D) Normalized frequencies of rPT for correct (red area) and incorrect trials (green area). In C and D bin width is 35 ms.

**
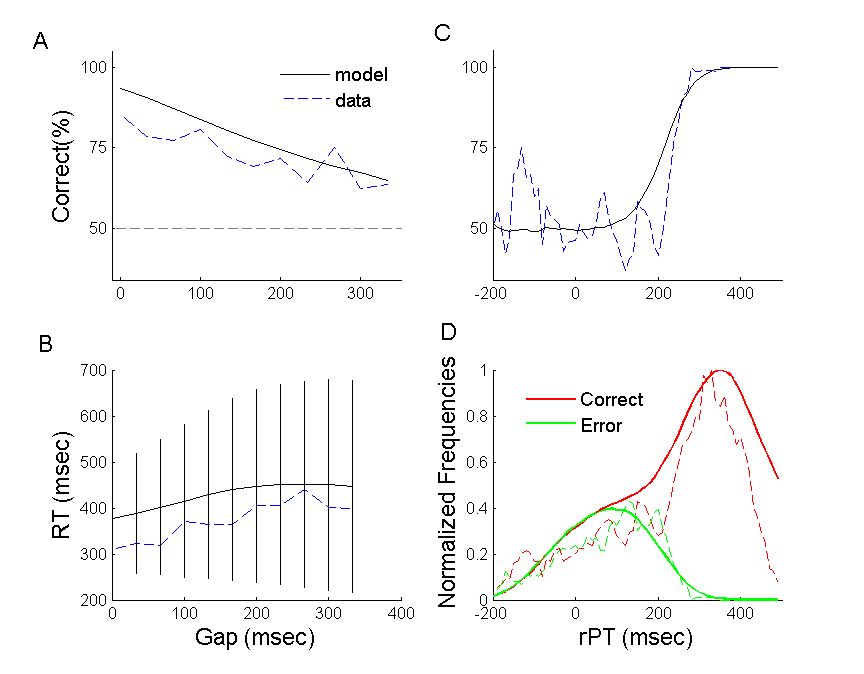
**

**Figure S6: Experiment 1, Individual fit of Participant 1 for Stanford et al model.** In A, B and C behavioral data are represented in dashed blue lines. Results labeled “model” are from simulated trials generated with best fitting parameters for Participant 1. (A) Percentage of correct responses as a function of time gap. (B) Mean reaction time (RT) with standard deviation for the whole data set as a function of time gap. (C) Percentage of correct responses as a function of raw Processing Time (rPT). (D) Normalized frequencies of rPT for correct (red area) and incorrect trials (green area). In C and D bin width is 35 ms.

**Supplementary References**

| 1. | Ratcliff, R. (1978). A theory of memory retrieval. *Psychological Review, 85*, 59-108. |
| --- | --- |
| 2. | Zhang, J., [Bogacz](http://www.cs.bris.ac.uk/Publications/pub_master.jsp?author=137549) ,R,(2010) [Bounded Ornstein-Uhlenbeck models for two-choice time controlled tasks](http://www.cs.bris.ac.uk/Publications/pub_master.jsp?id=2001156). *Journal of Mathematical Psychology*, *54*, 322–333. |
| 3. | Adam, R., Bays, P.M., Husain, M. (2012). Rapid decision-making under risk. *Cognitive Neuroscience*, *3*, 52-61. |
| 4. | Stanford, T. R., Shankar, S., Massoglia, D. P., & Salinas, E. (2010). Perceptual decision making in less than 30 milliseconds. *Nature Neuroscience, 13*(3), 379-385. |
